# Efficacy and safety of evolocumab in individuals with type 2 diabetes mellitus: primary results of the randomised controlled BANTING study

**DOI:** 10.1007/s00125-019-4856-7

**Published:** 2019-04-05

**Authors:** Robert S. Rosenson, Martha L. Daviglus, Yehuda Handelsman, Paolo Pozzilli, Harold Bays, Maria Laura Monsalvo, Mary Elliott-Davey, Ransi Somaratne, Peter Reaven

**Affiliations:** 1 Icahn School of Medicine at Mount Sinai, 1425 Madison Ave, MC Level, New York, NY 10029 USA; 2 University of Illinois at Chicago College of Medicine, Chicago, IL USA; 3 Metabolic Institute of America, Tarzana, CA USA; 4 University Campus Bio-medico, Rome, Italy; 5 Louisville Metabolic and Atherosclerosis Research Center, Louisville, KY USA; 6 Amgen Inc., Thousand Oaks, CA USA; 7 Amgen Ltd, Cambridge, UK; 8 University of Arizona College of Medicine, Phoenix VA Health Care System, Phoenix, AZ USA

**Keywords:** Diabetes, Diabetic dyslipidaemia, Hypercholesterolaemia, Lipid-lowering therapy, PCSK9 inhibition

## Abstract

**Aims/hypothesis:**

The study aimed to examine the efficacy of 12 weeks of monthly evolocumab or placebo in lowering LDL-cholesterol (LDL-C) in individuals with type 2 diabetes and hypercholesterolaemia or mixed dyslipidaemia and on a maximum-tolerated statin of at least moderate intensity.

**Methods:**

For this randomised, placebo-controlled outpatient study, eligible individuals were ≥18 years old with type 2 diabetes, HbA_1c_ <10% (86 mmol/mol), had been on stable pharmacological therapy for diabetes for ≥6 months and were taking a maximum-tolerated statin dose of at least moderate intensity. Lipid eligibility criteria varied by history of clinical cardiovascular disease. Participants were randomised 2:1 to evolocumab 420 mg s.c. or placebo. Randomisation was performed centrally via an interactive web-based or voice recognition system. Allocation was concealed using the centralised randomisation process. Treatment assignment was blinded to the sponsor study team, investigators, site staff and patients throughout the study. Co-primary endpoints were mean percentage change in LDL-C from baseline to week 12 and to the mean of weeks 10 and 12. Additional endpoints included LDL-C <1.81 mmol/l, LDL-C reduction ≥50% and other lipids. Exploratory analyses included percentage changes in fasting and post mixed-meal tolerance test (MMTT) lipoproteins and lipids, glucose metabolism variables and inflammatory biomarkers.

**Results:**

In total, 421 individuals were randomised and analysed, having received evolocumab (280 participants) or placebo (141 participants) (mean [SD] age 62 [8] years; 44% women; 77% white). Evolocumab decreased LDL-C by 54.3% (1.4%) at week 12 (vs 1.1% [1.9%] decrease with placebo; *p* < 0.0001) and by 65.0% (1.3%) at the mean of weeks 10 and 12 (vs 0.8% [1.8%] decrease with placebo; *p* < 0.0001); it also decreased non-HDL-cholesterol (HDL-C) by 46.9% (1.3%) at week 12 (vs 0.6% [1.8%] decrease with placebo) and by 56.6% (1.2%) at the mean of weeks 10 and 12 (vs 0.1% [1.6%] decrease with placebo). Evolocumab significantly improved levels of other lipids and allowed more participants to reach LDL-C <1.81 mmol/l or a reduction in LDL-C levels ≥50%. After an MMTT (120 min), there were favourable changes (*p* < 0.05; nominal, post hoc, no multiplicity adjustment) in chylomicron triacylglycerol (triglycerides), chylomicron cholesterol, VLDL-C and LDL-C. Evolocumab had no effect on glycaemic variables and was well tolerated.

**Conclusions/interpretation:**

In statin-treated individuals with type 2 diabetes and hypercholesterolaemia or mixed dyslipidaemia, evolocumab significantly reduced LDL-C and non-HDL-C. Favourable changes (*p* < 0.05) were observed in postprandial levels of chylomicrons, VLDL-C and LDL-C.

**Trial registration:**

ClinicalTrials.gov NCT02739984

**Funding:**

This study was funded by Amgen Inc.

**Data availability:**

Qualified researchers may request data from Amgen clinical studies. Complete details are available at www.amgen.com/datasharing.

**Electronic supplementary material:**

The online version of this article (10.1007/s00125-019-4856-7) contains peer-reviewed but unedited supplementary material, which is available to authorised users.

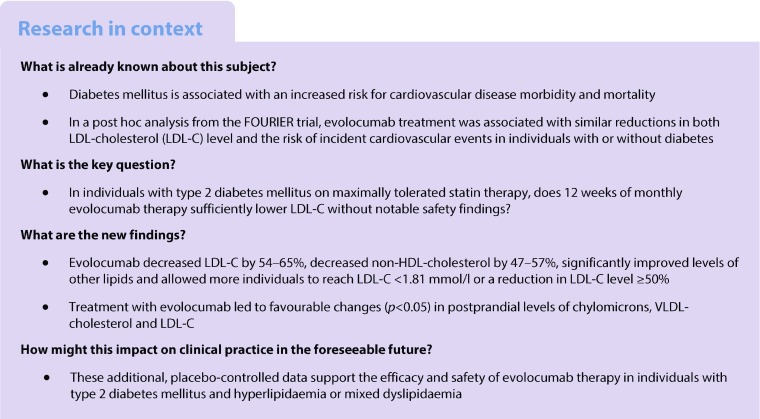



## Introduction

The prevalence of diabetes mellitus has increased progressively worldwide over the past several decades [1, 2], and diabetes mellitus is associated with an increased risk for cardiovascular disease (CVD) morbidity and mortality [3, 4]. Individuals with diabetes mellitus who experience an acute myocardial infarction (MI) are at higher risk for recurrent cardiovascular events and mortality compared with their counterparts without diabetes mellitus [3, 4], and thus require an aggressive treatment approach [5–7]. In addition, many statin-treated individuals with type 2 diabetes have poorly controlled LDL-cholesterol (LDL-C) and non-HDL-cholesterol (non-HDL-C) levels [7]. In the Further Cardiovascular Outcomes Research With PCSK9 Inhibition in Subjects With Elevated Risk (FOURIER) trial (ClinicalTrials.gov NCT01764633), the use of evolocumab 140 mg every 2 weeks or 420 mg once every month was associated with similar reductions in both LDL-C levels and the risk of incident cardiovascular events in individuals with or without diabetes [8].

The evolocumaB efficAcy aNd safeTy IN type 2 diabetes mellitus on backGround statin therapy (BANTING) study (NCT02739984) aimed to examine the efficacy of a 12 week regimen of s.c. evolocumab 420 mg once monthly compared with placebo in lowering LDL-C and improving other lipid levels in individuals with type 2 diabetes and hypercholesterolaemia or mixed dyslipidaemia taking maximally tolerated background statin therapy of at least moderate intensity.

## Methods

The primary objective of this study was to evaluate the effect of 12 weeks of treatment with s.c. monthly evolocumab compared with monthly placebo on percentage change in LDL-C among individuals with type 2 diabetes and hypercholesterolaemia or mixed dyslipidaemia on a maximally tolerated dose of statin of at least moderate intensity. The secondary objectives were to assess the effects of 12 weeks of treatment with monthly evolocumab compared with monthly placebo on: (1) change in LDL-C from baseline and the percentage change in non-HDL-C, apolipoprotein B (ApoB), total cholesterol, lipoprotein(a) (Lp(a)), triacylglycerol (triglycerides), HDL-C and VLDL cholesterol (VLDL-C); (2) percentage of participants achieving LDL-C <1.81 mmol/l; and (3) percentage of participants achieving a ≥50% reduction in LDL-C from baseline. The safety and tolerability of evolocumab were also assessed. Exploratory analyses included percentage changes in fasting, 120 min and 180 min post mixed-meal lipoprotein and glucose metabolism measures, and the inflammatory biomarkers adiponectin and IL-6.

Eligible individuals were ≥18 years of age with type 2 diabetes, had HbA_1c_ <10% (86 mmol/mol), were receiving stable pharmacological therapy for diabetes for ≥6 months, and were taking a maximally tolerated dose of statin of at least moderate intensity (per the American College of Cardiology [ACC]/American Heart Association [AHA] definition). Eligibility criteria for LDL-C or non-HDL-C level varied depending on prior clinical CVD, defined as a history of MI, stable or unstable angina, coronary or other arterial revascularisation, stroke, transient ischaemic attack or peripheral arterial disease presumed to be of atherosclerotic origin. Individuals without known clinical CVD were required to have a fasting LDL-C during lipid stabilisation of ≥2.59 mmol/l or non-HDL-C ≥3.39 mmol/l. Individuals with known clinical CVD were required to have a fasting LDL-C during lipid stabilisation of ≥1.81 mmol/l or non-HDL-C ≥2.59 mmol/l. Individuals were randomised 2:1 to two treatment groups (evolocumab and placebo). Randomisation was performed centrally via an interactive web-based or voice recognition system. Allocation was concealed using the centralised randomisation process. Treatment assignment was blinded to the sponsor study team, investigators, site staff, and patients throughout the study. Randomisation was stratified by LDL-C (above or below 3.36 mmol/l).

The co-primary endpoints were mean percentage change in LDL-C from baseline to week 12 and the mean percentage change in LDL-C from baseline to the mean of weeks 10 and 12. In this study, evolocumab was dosed at day 1, week 4 and week 8. Thus, week 12 was at the end of the dosing window. As maximum reduction of LDL-C occurs approximately 2 weeks post dose for individuals receiving evolocumab 420 mg monthly [9], LDL-C would be measured at week 10, before LDL-C levels began to return to baseline. Thus, an average measure between weeks 10 and 12 was also taken for lipid measures. Secondary lipid endpoints for these same time periods included: change from baseline in LDL-C, percentage change from baseline in non-HDL-C, ApoB, total cholesterol, Lp(a), triacylglycerol, HDL-C and VLDL-C; achievement of LDL-C <1.81 mmol/l; and ≥50% reduction in LDL-C from baseline.

Exploratory analyses included percentage changes in AUC in fasting, 120 min and 180 min mixed-meal tolerance test (MMTT) lipoproteins and lipids, glucose metabolism variables and inflammatory biomarkers. For the MMTT, following an overnight fast, participants were fed a standardised liquid mixed meal. Meals could differ between study sites but were required to contain the same amount (component weight not differing by more than ±15%) of energy (1004.16 kJ [240 kcal]), protein (10 g), total fat (4 g) and carbohydrate (41 g). The same type of standard mixed meal was required to be used for day 1 and week 12. Baseline and 2 h (±10 min) blood collection was done after the meal. In addition, a subset of individuals participated in MMTT extended-timepoint assessments, with three additional postprandial blood draws at 30 min (±10 min), 1 h (±10 min) and 3 h (±10 min). AUCs for 0–120 min and for 0–180 min were calculated for the MMTT at day 1 and at week 12 for laboratory variables. Lipid and lipoprotein measures included apolipoprotein B48 (ApoB-48), chylomicron triacylglycerol and cholesterol, total cholesterol, LDL-C, VLDL-C, non-HDL-C, triacylglycerol and HDL-C. Measures of glucose metabolism included plasma glucose, insulin, proinsulin, C-peptide, glucagon and NEFA. Anti- and proinflammatory biomarkers were adiponectin and IL-6, respectively.

LDL-C was calculated using the Friedewald formula; however, if calculated LDL-C was <1.03 mmol/l or triacylglycerol >4.52 mmol/l, then LDL-C was measured by ultracentrifugation from the same blood sample if additional adequate sample was available. VLDL-C was also calculated; similarly, if calculated LDL-C was <1.03 mmol/l or triacylglycerol >4.52 mmol/l, VLDL-C was quantified via ultracentrifugation. Chylomicron triacylglycerol and cholesterol were measured by photometry after ultracentrifugation. All routine lipids, lipoproteins and inflammatory biomarkers were assayed in serum samples by MedPace (Cincinnati, OH, USA and Leuven, Belgium). Serum glucose and HbA_1c_ were assessed as part of chemistry, which was performed by Q^2^ Solutions (Valencia, CA, USA and Livingston, Scotland, UK). An institutional review board or independent ethics committee reviewed and approved this study and the study amendment at each study centre. This amendment entailed minor language changes that provided clarification for study centres and is not expected to have altered results. The investigator collected informed consent from all participants before any screening procedures were performed. This study was conducted in accordance with International Council for Harmonisation Good Clinical Practice regulations/guidelines.

### Statistical analysis

**Fig. 1:**
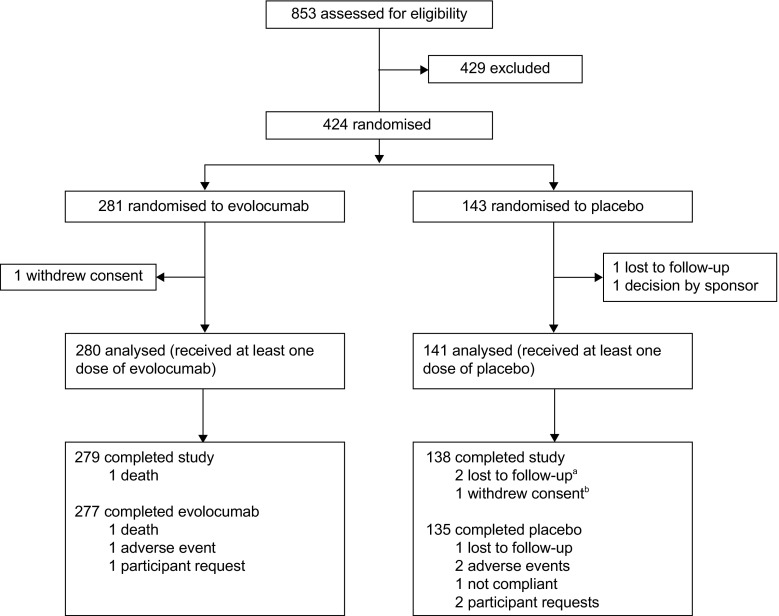
CONSORT flowchart of study design. Numbers of participants who completed the study and numbers who completed the course of evolocumab/placebo are shown separately. ^a^One of these participants completed placebo but was not reachable for the end of study visit; ^b^discontinued placebo with the reason of ‘participant request’

Safety and efficacy analyses were performed on all participants included in the full analysis set (all individuals who were randomised and received at least one dose of evolocumab or placebo; Fig. 1). A repeated-measures linear effects model was used to compare the efficacy of evolocumab vs placebo. This model includes terms for treatment group, pre-specified stratification by LDL-C level (<3.36 mmol/l vs ≥3.36 mmol/l), scheduled visit and the interaction of treatment with scheduled visit. Missing values were not imputed. A Cochran–Mantel–Haenszel test adjusted by stratification factor was used to analyse LDL-C level achievement and LDL-C response. For testing, non-response was imputed for individuals with a missing value.

The adjusted *p* values were calculated based on the multiplicity-testing strategy depicted in electronic supplementary material (ESM) Fig. 1; a *p* value of 0.05 was used for comparative purposes to determine statistical significance. An overall family-wise error rate of 0.05 was maintained for all co-primary and co-secondary efficacy outcome testing using a combination of sequential testing, the fall-back procedure and the Hochberg procedure. All analyses were performed using SAS version 9.4 (SAS Institute, Cary, NC, USA).

## Results

### Participants

A total of 421 individuals were randomised and received evolocumab (*n* = 280) or placebo (*n* = 141). The mean (SD) age was 62.5 (8.5) years for evolocumab and 62.2 (8.4) years for placebo; 42.9% (evolocumab) and 46.1% (placebo) of participants were female; 79.3% (evolocumab) and 71.6% (placebo) of participants were white (Table 1). Baseline clinical characteristics, including systolic BP, BMI, waist circumference and background lipid- and diabetes-related medication use were similar between evolocumab and placebo groups. The mean (SD) baseline LDL-C was 2.81 (0.80) mmol/l in the evolocumab treatment group and 2.86 (0.85) mmol/l in the placebo group. Non-HDL-C was 3.75 (0.90) mmol/l in the evolocumab group and 3.77 (0.88) mmol/l in the placebo group. Other lipids were also well matched at baseline between evolocumab and placebo treatment groups.

**Table 1:** Baseline demographics and characteristics of the study population

Characteristic	Placebo (*n*=141)	Evolocumab (*n*=280)
Demographic		
Sex, female, *n* (%)	65 (46.1)	120 (42.9)
Age, years, mean (SD)	62.2 (8.4)	62.5 (8.5)
Race, white, *n* (%)	101 (71.6)	222 (79.3)
Ethnicity, not Hispanic/Latino, *n* (%)	117 (83.0)	226 (80.7)
Clinical		
Systolic BP, mmHg, mean (SD)	131.4 (17.8)	129.9 (14.6)
BMI, kg/m^2^, mean (SD)	33.1 (7.2)	33.4 (6.1)
Waist circumference, cm, mean (SD)	108.9 (16.5)	109.0 (15.8)
Background lipid therapy per ACC/AHA definition, *n* (%)^a^		
High-intensity statin	72 (51.1)	146 (52.1)
Moderate-intensity statin	67 (47.5)	133 (47.5)
Hypertension, *n* (%)	119 (84.4)	247 (88.2)
Cerebrovascular or peripheral arterial disease, *n* (%)	33 (23.4)	67 (23.9)
Coronary artery disease, *n* (%)	44 (31.2)	119 (42.5)
Coronary artery stenosis >50%	23 (16.3)	51 (18.2)
Myocardial ischaemia	15 (10.6)	29 (10.4)
Angina pectoris	16 (11.3)	56 (20.0)
Myocardial infarction	22 (15.6)	46 (16.4)
Coronary artery bypass	13 (9.2)	45 (16.1)
Percutaneous coronary intervention	26 (18.4)	63 (22.5)
COPD, *n* (%)	10 (7.1)	24 (8.6)
Diabetes-related medication use, *n* (%)	141 (100)	280 (100)
Insulin use, *n* (%)	54 (38.3)	97 (34.6)
Lipid values, mean (SD)		
LDL-C, mmol/l, mean (SD)	2.86 (0.85)	2.81 (0.80)
Non-HDL-C, mmol/l, mean (SD)	3.77 (0.88)	3.75 (0.90)
ApoB, g/l, mean (SD)	0.98 (0.22)	0.97 (0.23)
Total cholesterol, mmol/l, mean (SD)	4.94 (0.91)	4.88 (0.95)
Lp(a), nmol/l, mean (SD)	99.40 (122.80)	88.00 (111.50)
Triacylglycerol, mmol/l, mean (SD)	2.00 (1.01)	2.08 (1.16)
HDL-C, mmol/l, mean (SD)	1.17 (0.32)	1.13 (0.33)
HbA_1c_, %, median (Q1, Q3)	7.2 (6.5, 8.2)	7.3 (6.5, 8.4)
HbA_1c_, mmol/mol, median (Q1, Q3)	55 (48, 66)	56 (48, 68)
Fasting serum glucose, mmol/l, median (Q1, Q3)	7.4 (6.0, 9.2)	7.7 (6.1, 9.6)

^a^Criteria modified from ACC/AHA guidelines: high intensity, atorvastatin 40–80 mg, rosuvastatin 20–40 mg, simvastatin 80 mg; moderate intensity, atorvastatin 10–20 mg, rosuvastatin 5–10 mg, simvastatin 20–40 mg, pravastatin 40–80 mg, lovastatin 40 mg, fluvastatin XL 80 mg, pitavastatin 2–4 mg

### Changes in lipids and glycaemic measures

Versus placebo, evolocumab treatment decreased LDL-C by a mean (SEM) of 53.1% (2.3%) at week 12 and by 64.1% (2.1%) at the mean of weeks 10 and 12 (combined *p* < 0.0001) (Tables 2 and 3). Compared with the placebo group, more participants in the evolocumab group achieved an LDL-C level <1.81 mmol/l (84.5% vs 15.4% at week 12, and 92.7% vs 14.8% at the mean of weeks 10 and 12; combined *p* < 0.0001). A ≥50% reduction in LDL-C was more common in the evolocumab group vs placebo group (65.5% vs 0.8% at week 12, and 84.2% vs 0.7% at the mean of weeks 10 and 12; combined *p* < 0.0001).

**Table 2:** Efficacy results at week 12 and at the mean of weeks 10 and 12

Variable	Week 12		Mean of weeks 10 and 12	
	Placebo (*n* = 141)	Evolocumab (*n* = 280)	Placebo (*n* = 141)	Evolocumab (*n* = 280)
LDL-C				
Change from baseline, %, mean (SEM)^a^	−1.1 (1.9)	−54.3 (1.4)	−0.8 (1.8)	−65.0 (1.3)
Treatment difference, mean (SEM)^b^	−53.1 (2.3)^†^		−64.1 (2.1)^†^	
Achievement of <1.81 mmol/l, *n* (%)	20 (15.4)	213 (84.5)	20 (14.8)	253 (92.7)
Achievement of ≥50% reduction, *n* (%)	1 (0.8)	165 (65.5)	1 (0.7)	221 (84.2)
Change from baseline in other lipids, %, mean (SEM)^a^				
Non-HDL-C	−0.6 (1.8)	−46.9 (1.3)	−0.1 (1.6)	−56.6 (1.2)
ApoB	1.8 (1.7)	−40.3 (1.3)	2.3 (1.6)	−50.2 (1.2)
Total cholesterol	−1.2 (1.4)	−35.0 (1.0)	−1.1 (1.2)	−42.2 (0.9)
Lp(a)	7.4 (3.1)	−25.2 (2.3)	9.6 (3.3)	−30.9 (2.4)
Triacylglycerol	4.8 (3.4)	−8.9 (2.5)	6.6 (2.9)	−12.6 (2.2)
HDL-C	−1.4 (1.4)	6.0 (1.0)	−2.6 (1.3)	7.2 (0.9)
VLDL-C	3.0 (2.9)	−10.3 (2.2)	3.4 (2.6)	−13.6 (1.9)
Change from baseline in glycaemic measure, median (Q1, Q3)				
HbA_1c_, %	0.1 (−0.2, 0.5)	0.1 (−0.2, 0.5)^‡^	N/A	N/A
HbA_1c_, mmol/mol	1.1 (−2.2, 5.5)	1.1 (−2.2, 5.5)		
Fasting serum glucose, mmol/l	0.2 (−0.8, 1.6)	0.3 (−0.8, 1.7)^§^	N/A	N/A

^a^Least-squares mean is from the repeated-measures model, which includes treatment group, stratification factor (from interactive voice response system), scheduled visit and the interaction of treatment with scheduled visit as covariates

^b^Treatment differences use s.c. placebo as the reference

^†^*p*<0.0001; ^‡^*p*=0.939; and ^§^*p*=0.785 all vs placebo

N/A, not applicable (not measured)

**Table 3:** Treatment difference between evolocumab vs placebo for lipid variables

Variable	Week 12		Mean of weeks 10 and 12	
Lipid efficacy, mean (95% CI) treatment difference				
Change from baseline in LDL-C, %	−53.1 (−57.6, −48.7)		−64.1 (−68.2, −60.1)	
Achievement of LDL-C <1.81 mmol/l, %	69.1 (60.4, 75.7)		77.9 (70.0, 83.5)	
Achievement of ≥50% reduction in LDL-C, %	64.7 (57.7, 70.3)		83.5 (77.7, 87.4)	
Per cent change from baseline in other lipids, mean (95% CI) treatment difference				
Non-HDL-C	−46.3 (−50.4, −42.2)		−56.6 (−60.3, −52.9)	
ApoB	−42.1 (−46.1, −38.1)		−52.5 (−56.1, −48.9)	
Total cholesterol	−33.7 (−36.9, −30.6)		−41.1 (−43.9, −38.2)	
Lp(a)	−32.6 (−39.6, −25.5)		−40.5 (−48.1, −32.9)	
Triacylglycerol	−13.7 (−21.6, −5.8)		−19.3 (−26.0, −12.5)	
HDL-C	7.4 (4.2, 10.6)		9.8 (7.0, 12.6)	
VLDL-C	−13.3 (−20.1, −6.6)		−17.1 (−22.9, −11.2)	

Placebo, *n*=141; evolocumab, *n*=280

All adjusted *p* values for measures reported in the table were *p*<0.0001 for evolocumab vs placebo comparison

The mean percentage change from baseline in other lipids is reported in Tables 2 and 3. Statistically significant improvements in favour of evolocumab were observed for non-HDL-C, ApoB, total cholesterol, Lp(a), triacylglycerol, HDL-C and VLDL-C (all *p* < 0.0001).

At week 12, fasting serum glucose measures changed from a median (quartile 1, quartile 3 [Q1, Q3]) baseline value of 7.7 (6.1, 9.6) mmol/l to a week 12 value of 8.2 (6.5, 10.0) mmol/l for evolocumab, and a median (Q1, Q3) baseline value of 7.4 (6.0, 9.2) mmol/l to a week 12 value of 7.8 (6.3, 10.3) mmol/l for placebo. These corresponded to median (Q1, Q3) changes from baseline to week 12 in fasting serum glucose of 0.3 (−0.8, 1.7) mmol/l for evolocumab-treated individuals, and 0.2 (−0.8, 1.6) mmol/l for placebo-treated individuals (Table 2).

At week 12, HbA_1c_ levels changed from a median (Q1, Q3) baseline value of 7.3% (6.5%, 8.4%) (56 [48, 68] mmol/mol) to 7.4% (6.7%, 8.6%) (57 [50, 70] mmol/mol) for evolocumab and from a median (Q1, Q3) baseline value of 7.2% (6.5%, 8.2%) (55 [48, 66] mmol/mol) to 7.4% (6.6%, 8.6%) (57 [49, 70] mmol/mol) for placebo. These corresponded to median (Q1, Q3) changes from baseline to week 12 in HbA_1c_ of 0.1% (−0.2%, 0.5%) (1.1 [−2.2, 5.5] mmol/mol) for evolocumab-treated participants and 0.1% (−0.2%, 0.5%) (1.1 [−2.2, 5.5] mmol/mol) for placebo-treated individuals.

### Changes in fasting and post-MMTT variables

For the exploratory endpoints of percentage change in fasting and postprandial lipid variables from day 1 to week 12 in response to MMTT, significant changes were observed in the evolocumab group for the mean or median AUC (0–120 min; Table 4). Results observed for the mean or median AUC for 0–180 min were consistent with those observed for 0–120 min, although not all variables were statistically significant; this may be due, in part, to almost complete clearance of chylomicrons by 120 min in some participants and the smaller sample sizes at 180 min (Table 4).

**Table 4:** Week 12 AUC for MMTT variables

Change from baseline to week 12 AUC (%)	AUC 0–120 min		AUC 0–180 min	
	Placebo	Evolocumab	Placebo	Evolocumab
Total cholesterol	1.7 (1.5) [128]	−30.9 (1.0)* [257]	1.1 (2.9) [27]	−31.3 (2.3) [57]
LDL-C	−2.1 (−10.5, 10.5) [117]	−60.6 (−71.6, −39.9)* [235]	−2.7 (−5.8, 3.8) [25]	−59.7 (72.8, −37.6)* [53]
HDL-C	−0.5 (1.1) [128]	8.7 (1.0)* [257]	4.0 (2.4) [27]	10.6 (2.3)* [57]
Non-HDL-C	2.7 (2.0) [128]	−42.7 (1.3) [257]	0.5 (3.7) [27]	−43.2 (2.8) [57]
Triacylglycerol	4.6 (−13.0, 29.3) [128]	−13.4 (−26.6, 8.4)* [257]	−4.7 (−18.7, 36.3) [27]	−17.0 (−32.7, 7.1)* [57]
VLDL-C	7.3 (3.0) [117]	−5.8 (2.4)* [239]	6.8 (6.8) [25]	−8.3 (4.4) [55]
Chylomicron triacylglycerol	6.9 (−19.9, 42.5) [96]	−12.8 (−33.8, 25.4)* [183]	−7.3 (−15.8, 23.0) [25]	−7.4 (−25.5, 26.1)* [44]
Chylomicron cholesterol	−1.8 (−16.7, 28.3) [97]	−19.7 (−34.1, 0.0)* [185]	−4.2 (−15.8, 28.3) [25]	−24.2 (−33.6, −5.4) [44]
ApoB-48	0.0 (−26.6, 31.6) [125]	−14.5 (−36.9, 18.2)* [252]	−0.7 (−26.2, 34.9) [26]	−8.5 (−36.3, 23.3) [59]
Plasma glucose	2.5 (−9.6, 22.8) [129]	2.6 (−9.0, 18.2) [257]	7.5 (−8.0, 18.8) [27]	6.6 (−8.5, 15.6) [60]
Insulin	6.6 (−22.5, 50.0) [126]	0.0 (−22.0, 29.1) [248]	14.3 (−14.1, 53.4) [27]	8.7 (−23.1, 40.6) [56]
Proinsulin	6.7 (−18.1, 41.6) [128]	0.0 (−22.4, 39.6) [260]	5.9 (−11.7, 49.1) [28]	−2.7 (−26.5, 19.9) [62]
C-peptide	4.1 (−13.8, 22.9) [125]	2.0 (−12.4, 17.0) [243]	−0.1 (−5.6, 30.1) [25]	2.4 (−18.4, 11.7) [55]
Glucagon	2.8 (−22.4, 39.2) [120]	−0.1 (−20.4, 23.4) [242]	0.5 (−18.9, 48.2) [25]	−3.3 (−23.3, 26.1) [57]
NEFA	−1.3 (−18.8, 21.3) [131]	−2.1 (−24.0, 26.7) [262]	2.2 (−26.8, 28.6) [28]	−4.6 (−19.6, 15.3) [61]
IL-6	−1.6 (−21.7, 17.4) [127]	−0.8 (−21.5, 29.5) [259]	3.8 (−11.4, 31.0) [28]	−5.8 (−25.7, 30.9) [59]
Adiponectin	−0.8 (−12.9, 12.3) [130]	−2.1 (−16.0, 15.0) [262]	1.7 (−11.6, 10.2) [28]	1.8 (−8.5, 17.7) [61]

All data presented are median (Q1, Q3) AUC [*n*], except for total cholesterol, HDL-C, non-HDL-C and VLDL-C, which are mean (SEM) AUC [*n*]

*n*, number of participants in the full analysis set who had MMTT timepoint (120 min) or extended-timepoint (180 min) assessments

*p<0.05 (nominal, post hoc, no multiplicity adjustment)

At week 12 after a mixed meal, the median (Q1, Q3) percentage change in AUC (0–120 min) for LDL-C was −60.6% (−71.6%, −39.9%) for evolocumab vs −2.1% (−10.5%, 10.5%) for placebo; and the mean (SEM) percentage change in AUC (0–120 min) for non-HDL-C was −42.7% (1.3%) for evolocumab vs 2.7% (2.0%) for placebo. The median (Q1, Q3) percentage change in AUC (0–120 min) was: for chylomicron cholesterol, −19.7% (−34.1%, 0.0%) for evolocumab vs −1.8% (−16.7%, 28.3%) for placebo; for chylomicron triacylglycerol, −12.8% (−33.8%, 25.4%) for evolocumab vs 6.9% (−19.9%, 42.5%) for placebo; and for ApoB-48, −14.5% (−36.9%, 18.2%) for evolocumab vs 0.0% (−26.6%, 31.6%) for placebo. The median (Q1, Q3) percentage change in AUC (0–120 min) for triacylglycerol was −13.4% (−26.6%, 8.4%) for evolocumab vs 4.6% (−13.0%, 29.3%) for placebo; mean (SEM) percentage change in AUC (0–120 min for VLDL-C was −5.8% (2.4%) for evolocumab vs 7.3% (3.0%) for placebo (Table 4). The aforementioned AUC (0–120) results were all significant with *p* < 0.05 for evolocumab.

For glucose metabolism measures in response to MMTT, median (Q1, Q3) AUC (0–120 min) were all non-significant: insulin (0.0% [−22.0%, 29.1%] for evolocumab vs 6.6% [−22.5%, 50.0%] for placebo); C-peptide (2.0% [−12.4%, 17.0%] for evolocumab vs 4.1% [−13.8%, 22.9%] for placebo); and glucagon (−0.1% [−20.4%, 23.4%] for evolocumab vs 2.8% [−22.4%, 39.2%] for placebo). Results for additional variables and AUC (0–180 min) can be found in Table 4.

Data are presented in ESM Table 1 for MMTT variables stratified by the baseline median triacylglycerol level of 1.8 mmol/l. Absolute values at week 12 for 0 min and 120 min timepoints are provided in ESM Table 2.

**Safety** Treatment-emergent adverse events (AEs) are listed in Table 5. AEs were reported in 110 (39.3%) evolocumab-treated participants and 52 (36.9%) placebo-treated participants. Serious AEs occurred in 14 (5.0%) evolocumab-treated participants and two (1.4%) placebo-treated participants. No serious AE was considered to be related to the investigational product or to the device. No pattern in the serious AE was identified in either group. Chronic obstructive pulmonary disease (COPD) was the only serious AE reported by ≥1% of participants in any treatment group (1.4% evolocumab, 0% placebo). AEs led to study discontinuation in 0.4% (evolocumab) and 1.4% (placebo) of participants; none of these AEs was considered serious. The most common AEs occurring in ≥2% of participants in either treatment group are shown in Table 5. AEs reported in ≥2% of participants in the evolocumab group and not in the placebo group were hypertension (3.9%) and COPD (2.1%). Ten of the 11 participants reporting an AE of hypertension had hypertension at baseline and were receiving pharmacological treatment (seven participants were treated with two or more drugs). Despite anti-hypertensive treatment, BP at baseline was ≥140 and/or 90 mmHg in eight participants reporting an AE of hypertension. Four of the six participants reporting an AE of COPD had COPD at baseline; two participants with a history of COPD were not receiving treatment at baseline; all six were current or former smokers.

**Table 5:** Safety

AE	Placebo (*n* = 141) *n* (%)	Evolocumab (*n* = 280) *n* (%)
Treatment-emergent	52 (36.9)	110 (39.3)
Serious^a^	2 (1.4)	14 (5.0)
Leading to discontinuation of evolocumab or placebo	2 (1.4)	1 (0.4)
Serious	0 (0.0)	0 (0.0)
Non-serious	2 (1.4)	1 (0.4)
Fatal AEs^b^	0 (0.0)	1 (0.4)
Most common^c^		
Hypertension	0 (0.0)	11 (3.9)
Diabetes mellitus^d^	5 (3.5)	8 (2.9)
Diarrhoea	4 (2.8)	6 (2.1)
Headache	3 (2.1)	6 (2.1)
Urinary tract infection	2 (1.4)	6 (2.1)
COPD	0 (0.0)	6 (2.1)
Viral upper respiratory tract infection	4 (2.8)	5 (1.8)
Pharyngitis	3 (2.1)	0 (0.0)
Back pain	3 (2.1)	2 (0.7)
Abnormal laboratory tests		
CK >5 × ULN	0 (0.0)	1 (0.4)^e^
CK >10 × ULN	0 (0.0)	1 (0.4)^e^
AST or ALT >3 × ULN	1 (0.7)	1 (0.4)

^a^COPD was the only AE reported by ≥ 1% of participants in any treatment group (1.4% evolocumab, 0% placebo). Four COPD, two coronary artery disease and one hypertension serious AE were preceded by the same disease history (i.e. the COPD event was preceded by history of COPD) at baseline. One participant in the evolocumab group experienced four serious AEs (COPD, bacterial pneumonia, sepsis and dehydration). One participant in the evolocumab group with a history of coronary artery disease, MI, peripheral arterial disease (PAD) and stroke experienced a fatal AE of sudden cardiac death; this event was not considered related to the investigational product by the investigator

^b^Sudden cardiac death 8 days after exposure to evolocumab; not considered related to evolocumab by investigator

^c^Reported in ≥ 2% of participants in one or more treatment groups

^d^Worsening of diabetes or diabetes control per investigator

^e^Participant reported vigorous exercise prior to the week 12/end of study visit

ALT, alanine aminotransferase; AST, aspartate aminotransferase; CK, creatine kinase; ULN, upper limit of normal

## Discussion

Many individuals with type 2 diabetes receiving statin therapy have LDL-C and non-HDL-C levels that exceed recommended lipid levels [10]. Persistent elevations in LDL-C and non-HDL-C may derive from insufficient LDL-C-lowering efficacy with moderate- to high-intensity statins, lack of statin titration in individuals with diabetes mellitus taking a low- to moderate-intensity statin who are not titrated to a high-intensity dosage [7] or statin-associated AEs [11]. In a high-risk population of individuals with diabetes who were treated with a high-intensity statin, higher VLDL-C and small cholesterol-enriched VLDL particles were associated with an increased risk of recurrent atherosclerotic cardiovascular disease (ASCVD) events [1].

We investigated the efficacy and safety of evolocumab in individuals with type 2 diabetes who had elevated LDL-C or non-HDL-C levels on a maximally tolerated dose of a statin of at least moderate intensity. Several guidelines advocate more aggressive LDL-C lowering to levels <1.81 mmol/l in individuals with ASCVD or <2.6 mmol/l in individuals with diabetes. In our study, addition of evolocumab to background statin therapy greatly reduced LDL-C levels vs placebo and enabled most individuals to reach LDL-C levels <1.81 mmol/l. The ACC/AHA has adopted a desired LDL-C-lowering efficacy of >50% [12], with an updated decision pathway recommending a >50% reduction in LDL-C and consideration of non-statin therapies such as ezetimibe or a proprotein convertase subtilisin/kexin type 9 inhibitor (PCSK9i) when additional LDL-C lowering is desired. Guidelines from the American Association of Clinical Endocrinologists (AACE) and ADA also recommend additional non-statin therapy for high-risk individuals who do not achieve an effective >50% reduction in LDL-C [2] or desired LDL-C levels [13, 14] with maximally tolerated statin. In the BANTING trial, we observed that a substantially greater proportion of participants randomised to evolocumab therapy vs placebo achieved ≥50% reduction in LDL-C. The shorter duration of follow-up (12 weeks) limited the ability to assess long-term safety and durability of response.

FOURIER included 11,031 (40%) participants with diabetes who received treatment with moderate- to high-intensity statin over a median follow-up of 26 months [8, 15]. The HR for the primary cardiovascular endpoint (cardiovascular death, MI, stroke, hospitalisation for unstable angina, or coronary revascularisation) in participants with diabetes was 0.83 (95% CI 0.75, 0.93; *p* = 0.0008) and 0.87 (0.79, 0.96; *p* = 0.0052) for participants without diabetes (*p*_interaction_ = 0.64) [8]. The HR for the secondary endpoint was 0.82 (0.72, 0.93; *p* = 0.0021) for individuals with diabetes and 0.78 (0.69, 0.89; *p* = 0.002) for those without diabetes [8]. Because of the higher baseline cardiovascular risk of individuals with type 2 diabetes, the absolute risk reduction in the primary endpoint with evolocumab tended to be greater in those with diabetes (2.7% [95% CI 0.7, 4.8]) vs those without diabetes (1.6% [0.1, 3.2]) over 3 years [8].

Non-HDL-C is more strongly associated with ASCVD events than LDL-C in participants with diabetes, and it is a co-primary or secondary target of cholesterol-lowering therapies [13, 14]. Here, in the BANTING study, we evaluated the efficacy of evolocumab therapy on non-HDL-C levels and on the achievement of non-HDL-C levels that are recommended by several guidelines. Evolocumab reduced non-HDL-C levels by 47% vs 1% reduction with placebo at week 12, and by 57% vs 1% reduction with placebo at the mean of weeks 10 and 12. Evolocumab also improved levels of other lipid and lipoprotein fractions.

No notable effects in response to MMTT were observed in the evolocumab treatment group for glucose metabolism measures (plasma glucose, insulin, proinsulin, C-peptide, glucagon and NEFA) (median AUC 0–120 min and/or median AUC 0–180 min) (Table 4). The reductions we observed in postprandial triacylglycerol-rich lipoproteins such as VLDL-C, chylomicron triacylglycerol and ApoB-48 were consistent with the concept that remnant lipoproteins are cleared, in part, by the LDL receptor. Our results differ from a recent analysis from the Effects on Lipoprotein Metabolism From PCSK9 Inhibition Utilizing a Monoclonal Antibody (FLOREY) study, where postprandial levels of chylomicrons and triacylglycerol were not altered in healthy men with LDL-C >2.59 mmol/l treated with evolocumab [16]. However, the FLOREY analysis was conducted in a small number of normolipidaemic men. Our results here are largely consistent with those of other phase II and III studies of evolocumab, demonstrating that evolocumab treatment leads to modest reductions in triacylglycerol [17, 18].

In this study of participants with type 2 diabetes, there were no notable differences in fasting glucose or HbA_1c_ levels between treatment arms at the end of study. At 2 h after a mixed meal, there were no differences between treatment arms in glucose, insulin, proinsulin, C-peptide, glucagon or NEFA concentrations. These data support the short-term safety of evolocumab on glycaemic variables in individuals with type 2 diabetes.

In FOURIER, levels of HbA_1c_ and fasting plasma glucose were similar over time in both the evolocumab and placebo groups in participants with or without diabetes, and evolocumab did not increase the risk of new-onset diabetes in participants without diabetes (HR 1.05; 95% CI 0.94, 1.17), including those participants with impaired fasting glucose at baseline (HR 1.00; 95% CI 0.89, 1.13). In participants with diabetes at baseline, incidences of AEs and serious AEs were similar between placebo and evolocumab treatment groups in FOURIER, and imbalances between these treatment groups were not observed for the incidence of hypertension or COPD AEs [8]. The safety and efficacy of the PCSK9i alirocumab in individuals with diabetes has been evaluated in several multicentre trials. Safety and changes in lipid efficacy were similar in individuals with insulin-treated diabetes, regardless of diabetes type [19], and alirocumab had no effect on the transition to new-onset diabetes in individuals without diabetes at baseline [20, 21]. These reports have not indicated any imbalances between treatment groups in the incidence of hypertension or COPD.

In conclusion, in statin-treated individuals with type 2 diabetes and hypercholesterolaemia or mixed dyslipidaemia, the addition of evolocumab compared with placebo resulted in LDL-C reductions of up to 64% and LDL-C levels <1.81 mmol/l in up to 93% of individuals. In this patient population, the addition of evolocumab compared with placebo resulted in non-HDL-C reductions of up to 57%. Evolocumab treatment had no notable effect on glucose measures and was safe and well tolerated overall.

## Electronic supplementary material

ESM (PDF 164 kb)

## Data Availability

Qualified researchers may request data from Amgen clinical studies. Complete details are available at www.amgen.com/datasharing.

## Abbreviations

ACC: American College of Cardiology
AE: Adverse event
AHA: American Heart Association
ApoB: Apolipoprotein B
ApoB-48: Apolipoprotein B48
ASCVD: Atherosclerotic cardiovascular disease
BANTING: EvolocumaB efficAcy aNd safeTy IN type 2 diabetes mellitus on backGround statin therapy
COPD: Chronic obstructive pulmonary disease
CVD: Cardiovascular disease
FOURIER: Further Cardiovascular Outcomes Research With PCSK9 Inhibition in Subjects With Elevated Risk
HDL-C: HDL-cholesterol
LDL-C: LDL-cholesterol
Lp(a): Lipoprotein(a)
MI: Myocardial infarction
MMTT: Mixed-meal tolerance test
PCSK9i: Proprotein convertase subtilisin/kexin type 9 inhibitor
Q: Quartile
VLDL-C: VLDL cholesterol
