# Supplementary material for: Efficacy and safety of evolocumab in individuals with type 2 diabetes mellitus: primary results of the randomised controlled BANTING study
Source: Diabetologia. 2019 Apr 5;62(6):948–58. doi: 10.1007/s00125-019-4856-7 (PMC6509076; doi:10.1007/s00125-019-4856-7)
Supplement: Supplementary file 1 — (PDF 164 kb) [file 125_2019_4856_MOESM1_ESM.pdf]

ELECTRONIC SUPPLEMENTARY MATERIAL

Efficacy and safety of evolocumab in individuals with type 2 diabetes mellitus: primary results of the randomised controlled BANTING study

Robert S. Rosenson, Martha L. Daviglus, Yehuda Handelsman, Paolo Pozzilli, Harold Bays, Maria Laura Monsalvo, Mary Elliott-Davey, Ransi Somaratne, Peter Reaven

ESM Table 1 Percent change from baseline to week 12 in AUC stratified by baseline triglyceride levels (Baseline median triglyceride: 1.8 mmol/L)

	Percent change from baseline to week 12 in AUC (mean, SEM)			
	**Baseline TG <1.8 mmol/L**		**Baseline TG ≥ 1.8 mmol/L**	
	Placebo	Evolocumab	Placebo	Evolocumab
**Total Cholesterol**	n=64	n=124	n=64	n=133
	**1.1 (2.2)**	**-31.1 (1.3)**	**2.2 (2.2)**	**-30.7 (1.6)**
**LDL-C**	n=64	n=123	n=53	n=112
	**0.9 (3.2)**	**-54.9 (2.0)**	**1.2 (3.1)**	**-53.5 (2.6)**
**HDL-C**	n=64	n=124	n=64	n=133
	**-2.5 (1.6)**	**5.4 (1.3)**	**1.6 (1.6)**	**11.7 (1.5)**
**Non–HDL-C**	n=64	n=124	n=64	n=133
	**2.7 (3.0)**	**-44.3 (1.8)**	**2.7 (2.7)**	**-41.1 (1.9)**
**Triglycerides**	n=64	n=124	n=64	n=133
	**15.7 (4.5)**	**0.9 (4.2)**	**3.8 (4.0)**	**-11.0 (3.2)**
**VLDL-C**	n=64	n=123	n=53	n=116
	**15.6 (4.5)**	**-1.6 (3.2)**	**-2.7 (3.3)**	**-10.3 (3.4)**
**Chylomicron triglycerides**	n=47	n=91	n=49	n=92
	**29.1 (10.2)**	**19.7 (9.7)**	**6.2 (7.0)**	**1.8 (8.4)**
**Chylomicron cholesterol**	n=48	n=92	n=49	n=93
	**8.5 (4.9)**	**-8.9 (3.8)**	**9.1 (6.0)**	**-18.3 (2.4)**
**ApoB48**	n=62	n=121	n=63	n=131
	**15.6 (5.7)**	**10.8 (7.7)**	**9.9 (8.3)**	**-7.5 (4.4)**

Abbreviations: ApoB48, apolipoprotein B48; AUC, area under the curve; HDL-C, high-density lipoprotein cholesterol; LDL-C, LDL cholesterol; n, number of patients in the full analysis set who had AUC assessments; TG, triglycerides; VLDL-C, VLDL cholesterol

ESM Table 2 Summary of Week 12 Absolute Values by MMTT Timepoints

	**Week 12 at 0 minutes**		**Week 12 at 120 minutes**	
	Placebo	Evolocumab	Placebo	Evolocumab
**Total Cholesterol**	n=134	n=269	n=133	n=269
(mmol/l)	**4.88 (0.09)**	**3.30 (0.06)**	**4.83 (0.09)**	**3.26 (0.06)**
**LDL-C**	n=124	n=256	n=123	n=253
(mmol/l)	**2.7 (2.1, 3.4)**	**1.2 (0.8, 1.6)**	**2.5 (2.0, 3.4)**	**1.1 (0.7, 1.5)**
**HDL-C**	n=134	n=269	n=133	n=269
(mmol/l)	**1.16 (0.03)**	**1.19 (0.02)**	**1.12 (0.03)**	**1.17 (0.02)**
**Non–HDL-C**	n=134	n=269	n=133	n=269
(mmol/l)	**3.73 (0.09)**	**2.11 (0.06)**	**3.71 (0.09)**	**2.10 (0.05)**
**Triglycerides**	n=134	n=269	n=133	n=269
(mmol/l)	**1.7 (1.3, 2.5)**	**1.5 (1.1, 2.2)**	**1.9 (1.5, 2.8)**	**1.8 (1.3, 2.5)**
**VLDL-C**	n=124	n=260	n=123	n=258
(mmol/l)	**0.83 (0.03)**	**0.78 (0.02)**	**0.92 (0.03)**	**0.87 (0.02)**
**Chylomicron triglycerides**	n=117	n=217	n=115	n=214
(mmol/l)	**0.42 (0.27, 0.67)**	**0.40 (0.25, 0.66)**	**0.59 (0.36, 0.88)**	**0.53 (0.35, 0.80)**
**Chylomicron cholesterol**	n=117	n=219	n=115	n=216
(mmol/l)	**0.88 (0.73, 1.04)**	**0.70 (0.60, 0.88)**	**0.93 (0.80, 1.11)**	**0.70 (0.60, 0.91)**
**ApoB48**	n=132	n=267	n=131	n=265
(g/l)	**0.01 (0.006, 0.015)**	**0.008 (0.006, 0.013)**	**0.012 (0.009, 0.019)**	**0.012 (0.008, 0.016)**
**Plasma glucose**	n=134	n=271	n=134	n=270
(mmol/l)	**8.0 (6.3, 10.7)**	**8.4 (6.5, 10.8)**	**10.7 (8.1, 14.3)**	**10.7 (8.0, 13.5)**
**Insulin**	n=133	n=266	n=133	n=264
(pmol/l)	**117.4 (75.7, 191.7)**	**129.9 (72.2, 211.8)**	**254.2 (149.3, 385.5)**	**231.3 (141.3, 416.4)**
**Pro-insulin**	n=133	n=268	n=132	n=268
(pmol/l)	**21.5 (12.3, 40.2)**	**28.0 (13.8, 51.5)**	**47.1 (29.8, 75.9)**	**51.4 (28.4, 80.9)**
**C-peptide**	n=133	n=264	n=133	n=263
(nmol/l)	**1.10 (0.74, 1.49)**	**1.25 (0.81, 1.71)**	**1.96 (1.33, 2.56)**	**1.98 (1.34, 2.67)**
**Glucagon**	n=128	n=264	n=128	n=264
(ng/l)	**173.5 (110.1, 233.6)**	**153.3 (109.1, 231.0)**	**191.8 (127.7, 265.7)**	**180.4 (127.6, 245.0)**
**Free fatty acids**	n=134	n=270	n=134	n=270
(mmol/l)	**0.57 (0.42, 0.71)**	**0.51 (0.38, 0.67)**	**0.32 (0.21, 0.44)**	**0.31 (0.22, 0.44)**
**Interleukin-6**	n=133	n=269	n=133	n=269
(pg/ml)	**2.9 (2.1, 4.9)**	**3.4 (2.3, 5.6)**	**2.7 (1.9, 4.3)**	**3.1 (2.0, 4.9)**
**Adiponectin**	n=132	n=268	n=132	n=267
(mcg/ml)	**5.0 (3.4, 6.6)**	**4.4 (3.0, 7.1)**	**5.0 (3.3, 6.5)**	**4.5 (3.0, 7.0)**

All data presented are median (Q1, Q3), except for total cholesterol, HDL-C, non-HDL-C, and VLDL-C, which are mean (SEM).

ESM Figure 1. Multiplicity Adjustment Methods

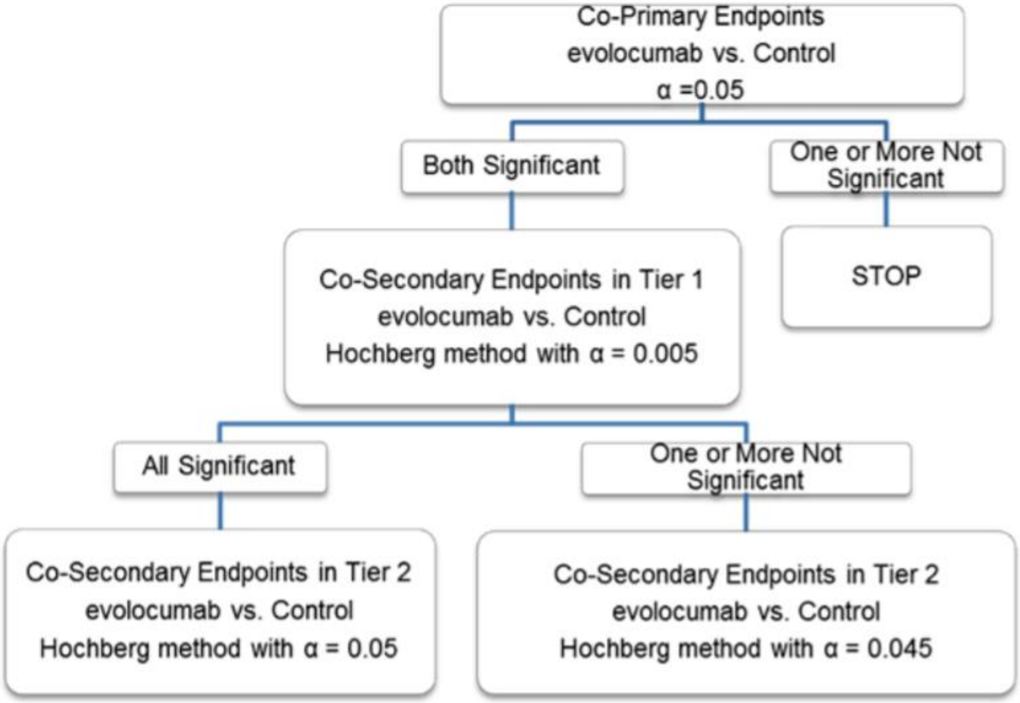

Tier 1 co-secondary endpoints: Change from baseline in LDL-C; percent change from baseline in non-HDL-C; percent change from baseline in ApoB, percent change from baseline in TC; achievement of target LDL-C <1.8 mmol/L; 50% reduction of LDL-C from baseline
Tier 2 co-secondary endpoints: Percent change from baseline in Lp(a); percent change from baseline in triglycerides; percent change from baseline in HDL-C; percent change from baseline in VLDL-C

Site Investigators by Country

Below is the list of principal investigators who have screened at least one patient.

Belgium: Ann Mertens (Leuven), André Scheen (Liege), Luc Van Gaal (Edegem).

Canada: Thomas Elliott (Vancouver), Pavel Hamet (Montreal), Yves Pesant (St-Jérôme), Eric St Amour (Gatineau).

Italy: Enzo Bonora (Verona), Andrea Palermo (Roma), Paolo Pozzilli (Roma).

Mexico: Luis Alejandro Nevarez Ruiz (Chihuahua).

Poland: Zbigniew Gaciong (Warszawa), Mateusz Sidor (Krakow), Krzysztof Strojek (Katowice), Malgorzata Zolcinska- Wilczynska ((Wroclaw).

Spain: Nuria Alonso Pedrol (Badalona), Ramon Gomis de Barbara (Barcelona), Pedro Mezquita Raya (Almeria), Manuel Muñoz Torres (Granada).

United States of America: Rafik Abadier (Inverness, FL), Stephan Babirak (Scarborough, MA), Darlene Bartilucci (Jacksonville, FL), Seth Baum (Boca Raton, FL), Harold Bays (Louisville, KY), Barry Bertolet (Tupelo, MS), Tira Chaicha-Brom (Austin, TX), Mark Christiansen (Walnut Creek, CA), Edmund Claxton (Auburn, ME), Lisa Connery (Norman, OK), Martha Daviglus (Chicago, IL), William Davila (Fleming Island), Juan Frias (Los Angeles, CA), Ronald Goldberg (Miami, FL), Stephen Grubb (Tabor City, NC), Yehuda Handelsman (Tarzana, CA), Terence Hart (Muscle Shoals, AL), Steven Hearne (Salisbury, MD), David Henderson (Daytona Beach, FL), Barry Horowitz (West Palm Beach, FL), Stephen Jones (Shelby, NC), Mark Kutner (Miami, FL), Dennis Levinson (Chicago, IL), Robert Lipetz (Spring Valley, CA), Sashi Makam (New Windsor, NY), Michael McCartney (Methuen, MA), Alan Miller (Dunwoody, GA), Paul Miller (Lakewood, CO), Lubna Mirza (Norman, OK), Samer Nakhle (Las Vegas, NV), Larry Odekirk (Denver, CO), Marina Raikhel (Lomita, CA), David Ramstad (Suffolk, VA), Peter Reaven (Phoenix, AZ), Jakkidi Reddy (Roseville, CA), Jeffrey Rosen (Coral Gables, CA), Robert Rosenson (New York, NY), Lance Rudolph (Albuquerque, NM), Aditya Samal (Houston, TX), Yshay Shlesinger (San Ramon, CA), Arthur Schwartzbard (New York, NY), Maria Solano (Miami, FL), Glen Sussman (Chicago, IL), Luis Tami (Hollywood, FL), Mark Turner (Meridian, ID), Howard Weintraub (New York, NY), Robert Weiss (Auburn, ME).
